# Role of Steroid Hormones in the Pathogenesis of Nonalcoholic Fatty Liver Disease

**DOI:** 10.3390/metabo11050320

**Published:** 2021-05-17

**Authors:** Meng Yang, Feng Ma, Min Guan

**Affiliations:** 1 Guangdong Provincial Key Laboratory of Medical Molecular Diagnostics, Institute of Biochemistry and Molecular Biology, Institute of Aging Research, Guangdong Medical University, Dongguan 523808, China; yangmeng116@126.com; 2 Center for Human Tissues and Organs Degeneration, Institute of Biomedicine and Biotechnology, Shenzhen Institute of Advanced Technology, Chinese Academy of Sciences, Shenzhen 518055, China; mf56718799@126.com

**Keywords:** NAFLD, steroid hormone, steroid hormone receptor

## Abstract

Nonalcoholic fatty liver disease (NAFLD) is the most common cause of chronic liver disease and may progress to cirrhosis or even hepatocellular carcinoma. A number of steroid hormones are important regulators of lipid homeostasis through fine tuning the expression of genes related to lipid synthesis, export, and metabolism. Dysregulation of such pathways has been implicated in the pathogenesis of NAFLD. The aim of this review is to clarify the potential impact of steroid hormones on NAFLD. We also highlight potential interventions through modulating steroid hormone levels or the activities of their cognate receptors as therapeutic strategies for preventing NAFLD.

## 1. Introduction

Nonalcoholic fatty liver disease (NAFLD) has been increasingly recognized as a worldwide health issue and is estimated to affect 20–30% of the general population [1]. It is a progressive disease ranging from steatosis to nonalcoholic steatohepatitis (NASH), which is a risk for the development of cirrhosis and hepatocellular carcinoma (HCC) [2,3]. Most patients with NAFLD have simple steatosis in the absence of hepatocyte injury. However, approximately 10–30% of patients with NAFLD develop NASH characterized by inflammation and fibrosis [4]. Over the last decade, it has been shown that NAFLD is a multisystem disease, affecting several extrahepatic organs and regulatory pathways [5]. NAFLD is tightly associated with obesity, metabolic syndrome, and type 2 diabetes [6]. All of these complications related to NAFLD pose significant health and economic burdens on patients and society.

The pathogenesis of NAFLD is complex and described by a “multiple-hit” hypothesis, which has been proposed to supersede the outdated “two-hit” hypothesis [7]. The “multiple-hit” hypothesis highlights the importance of genetic and epigenetic factors, nutritional factors, intestinal microbiota, insulin resistance, and hormones secreted from adipose tissue [7,8]. All forms of NAFLD tightly correlate with hepatic and peripheral insulin resistance in epidemiological, experimental, and human studies [9,10,11]. Insulin resistance upregulates de novo lipogenesis, inhibits β-oxidation of free fatty acids (FFAs) and increases the degradation of very-low-density lipoprotein (VLDL) in the liver, leading to an increase in hepatic fat accumulation [12,13,14]. It also promotes lipolysis rates in adipose tissue, leading to more fatty-acid efflux to the liver [15]. The intestinal microbiota has been found to alter bile acid metabolism to affect hepatic lipid handling and alter host immunity contributing to NASH [16]. There is increasing evidence reporting adiponectin as an adipokine with anti-inflammatory and antifibrogenic activity protecting liver parenchyma against steatosis and apoptosis [17].

Lipid accumulation within hepatocytes caused by these risk factors leads to steatosis, defined as the presence of fat comprising 5% of liver weight [18]. Exaggerated lipid accumulation can cause toxicity via diverse mechanisms. For instance, toxic lipid metabolites derived from FFAs impose oxidative stress on hepatocytes [19]. Furthermore, oxidative stress affects the function of the endoplasmic reticulum and mitochondria, as well as contributes to hepatic inflammation and fibrosis [20].

Steroid hormones have been discovered to play important roles in lipid metabolism. Deregulation of steroid hormone level or bioactivity has been implicated in various degree of hepatocellular damages. In Cushing’s syndrome, the high circulating steroid hormone (glucocorticoid) level causes visceral obesity, insulin resistance, and hepatic steatosis [21]. Activation of the renin–angiotensin–aldosterone system modulated by multiple steroid hormones and corresponding receptors causes liver inflammation and fibrosis [22,23]. Moreover, steroid hormone receptor-regulated target genes are involved in both cholesterol and fatty-acid metabolism [24] and, therefore, have been implicated in the pathogenesis of NAFLD.

In this review, we discuss the roles of steroid hormones in the pathogenesis of NAFLD. It is widely known that steroid hormones have crucial clinical and therapeutic implications in many diseases. Such knowledge may provide insight to develop novel therapy strategies for NAFLD.

## 2. Steroid Hormones and Cognate Receptors

Steroid hormones act as chemical messengers in the body and regulate a wide variety of physiological processes including development, growth, reproduction, and metabolism. They are all derived from cholesterol via a sequential series of enzyme-catalyzed reactions. Further modifications of steroid structure and function can occur in many tissues of the body such as the liver and brain [25]. The main classes of steroid hormones include sex hormones, glucocorticoids, mineralocorticoids, and vitamin D. Sex steroid hormones can be divided into three classes: estrogens, androgens, and progestins. At the cellular level, steroid hormones mediate their physiologic effects through binding to their cognate receptors, most of which are nuclear receptors. Once the hormone is bound to its receptor, dimerization of the receptors occurs [26]. Following the interaction of the steroid hormone–receptor complex with target genes, coregulators are recruited for activation or suppression of specific genes. Mutation or aberrant expression of steroid hormone receptors or coregulators affects the normal function of steroid hormones and leads to the development of diseases [27].

### 2.1. Steroid Hormones

#### 2.1.1. Estrogens

Estrogens are the main female sex steroid hormones associated with reproductive organs and responsible for the development of female sexual characteristics [28]. Estrogen (E1), estradiol (E2 or 17β-estradiol), and estriol (E3) are the three major forms of physiological estrogens [29].During the menstrual cycle, estrogen levels produced by the gonads fluctuate in response to the changes in follicle-stimulating hormone and pituitary luteinizing hormone. Estrogen and its derivatives have been heavily researched for their clinical applications in postmenopausal women for reducing the risk of coronary arterial disease, osteoporosis, and mortality [30]. Estrogen and its receptors modulate pathological processes including breast and endometrial cancer [31]. They also perform important functions in the secretion of sebum [32], deposition of fat, lipogenesis, and insulin sensitivity [33,34,35].

#### 2.1.2. Androgens

Androgens are the primary male sex steroid hormones that exert significant effects on male sexual and reproductive function [36]. Androgens belong to a group of hormones that include testosterone, dihydrotestosterone (DHT), dehydroepiandrosterone (DHEA), androstenedione, androstenone, and androstenediol. They play essential roles mainly through androgen receptor (AR), which is an important member of the nuclear receptor superfamily [37]. Androgens also seem to influence the development and growth of other organs, such as skeletal muscle, liver, and kidney [38]. Moreover, it has been reported that androgens are beneficial for improving the cognition in the elderly population [39].

#### 2.1.3. Progestogens

Progestogens are also a class of steroid hormones that bind to progesterone receptor (PR) [40]. Progesterone is the major and most important progestogen in the body. It is considered to play essential roles in both the female and the male reproductive system [41]. Specifically, progesterone displays essential roles in the maintenance of pregnancy, regulation of menstrual cycle, and preparation of the mammary glands for lactation and breastfeeding after parturition in women, whereas progesterone regulates testosterone synthesis, spermiogenesis, and sperm capacitation in men. In addition, progestogens were shown to be used in menopausal hormone therapy and transgender hormone therapy for transgender women [42].

#### 2.1.4. Glucocorticoids

Synthesized in the adrenal cortex, glucocorticoids are a class of steroid hormones that bind to glucocorticoid receptor (GR) [43]. Glucocorticoids such as cortisol play roles in mediating stress-related metabolic regulation, as well as immune modulation. It is one of the most potent anti-inflammatory compounds known, and it is used in treating diseases caused by an overactive immune system [44]. An activated glucocorticoid receptor–glucocorticoid complex suppresses inflammation by preventing the translocation of proinflammatory transcription factors from the cytoplasm into the nucleus [45]. Glucocorticoids are also of key significance in fetal development and body fluid homeostasis [46]. Glucocorticoids stimulate protein degradation in muscle, skin, and lymphoid tissue; consequently, the released amino acids can be used for glucose and glycogen synthesis [47]. Glucocorticoids seem to be involved in mitochondrial RNA and protein synthesis, regulating mitochondrial respiration and oxidative phosphorylation [48].

#### 2.1.5. Mineralocorticoids

Mineralocorticoids belong to another class of steroid hormones produced in the adrenal cortex, which are important in the regulation of extracellular volume homeostasis [49,50]. As the primary mineralocorticoid, aldosterone regulates the balance of salt and water. Blood volume and pressure are increased by aldosterone, which mainly promotes active reabsorption of sodium and passive reabsorption of water, as well as the active secretion of potassium into the principal cells of the cortical collecting tubule in the kidney [51]. Aldosterone increases blood pressure, and activation of the mineralocorticoid receptor (MR) can affect cardiac function via induction of inflammation and fibrosis [52]. Moreover, MR is reportedly associated with adipocyte dysfunction and vascular abnormalities, which may lead to obesity and insulin resistance [53,54,55].

#### 2.1.6. Vitamin D

Vitamin D is recognized as a class of steroid hormone. Among all types of vitamin D, vitamin D3 (known as cholecalciferol) and vitamin D2 (ergocalciferol) are the most important forms [56]. Cholecalciferol can be produced in the skin exposed to ultraviolet (UV) rays from sunlight. The ingestion of cholecalciferol and ergocalciferol from the diet is also a source of vitamin D [57]. The active form, calcitriol, which takes effects via vitamin D receptor (VDR), is responsible for increasing the absorption of magnesium, calcium, and phosphate [58]. It is essential in calcium homeostasis and metabolism affecting bone growth and remodeling. Moreover, calcitriol has been reported to attenuate systemic inflammation and plays a protective role in liver structure [59].

### 2.2. Steroid Hormone Receptors

Steroid receptors are critical for normal liver function, while NAFLD is associated with inappropriate transcriptional regulation by steroid receptors. The classical steroid hormone receptor family includes estrogen receptor (ER), AR, PR, GR, MR [60,61], and VDR. It is well known that most of these receptors are nuclear receptors. Nuclear receptors are transcription factors which sense changing environmental or hormonal signals and regulate gene expression. To support this function, all nuclear receptors share the same basic structure: an amino-terminal transcriptional activation domain containing activating function 1 (AF-1); a DNA-binding domain; a hinge region; a carboxy-terminal ligand-binding domain containing AF-2 [62]. Following the binding of steroid hormones, all these regions contribute to the changes in gene expression occurring through the combined action of coregulators and chromatin modifiers.

**Figure 1:**
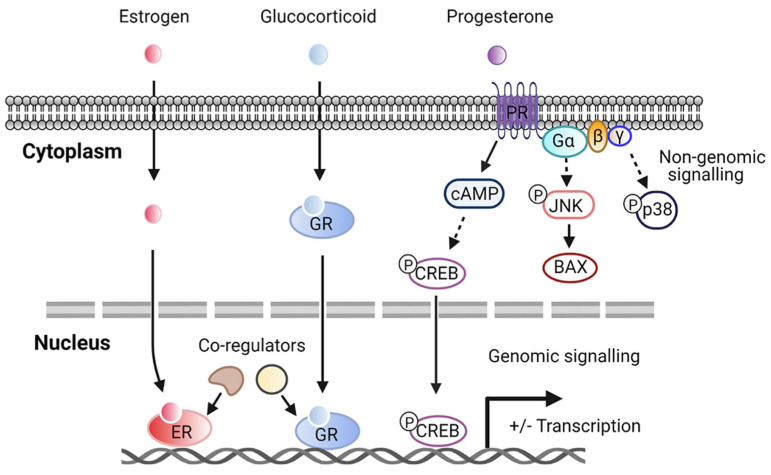
Representative steroid hormone genomic and nongenomic pathways. Abbreviations: estrogen receptor (ER); glucocorticoid receptor (GR); progesterone receptor (PR); cyclic adenosine monophosphate (cAMP); cAMP responsive element-binding protein (CREB); JUN N-terminal kinase (JNK); BCL2-associated X (BAX).

Although steroid hormone receptors are notably nuclear receptors, it has been reported that different steroid receptors in fact exist in extranuclear cellular pools. Steroid hormones may enter cells through the plasma membrane and bind to their receptors localized in the cytoplasm before translocating to the nucleus to regulate the transcription of target genes. In addition, new pathways of steroid hormones signaling through cell surface receptors contribute to more rapid, “non-nuclear”, or non-genomic signaling. Activated steroid receptors in the plasma membrane can stimulate second messenger cascades, interacting with several signaling molecules such as phosphatidylinositol 3-kinase (PI3K)/Akt, Ras/Raf-1, protein kinase A (PKA), and protein kinase C (PKC), leading to cell proliferation [63]. The molecular function of steroid hormones depends on the subcellular distribution of the receptors (Figure 1). Some sex hormone receptors, such as estrogen receptor α and β (ERα, ERβ), are found mainly in the nucleus [64,65]. Glucocorticoid receptors are predominantly localized in the cytoplasm and translocate to the nucleus upon binding to the ligand [66]. Recent evidence has demonstrated that membrane PRs mediate most of the nongenomic signaling of progesterone actions [67]. Progesterone-bound membrane PR potentiates cAMP production and cAMP responsive element-binding protein (CREB)-mediated transcription [68]. Furthermore, the activation of G protein via membrane PR induces the activation of JNK and p38 signaling pathways [67,68]. For novel therapeutic targets under consideration, priority should be given to these steroid receptors in nuclear or plasma membranes.

## 3. Role of Steroid Hormones in Hepatic Steatosis and Metabolism

**Figure 2:**
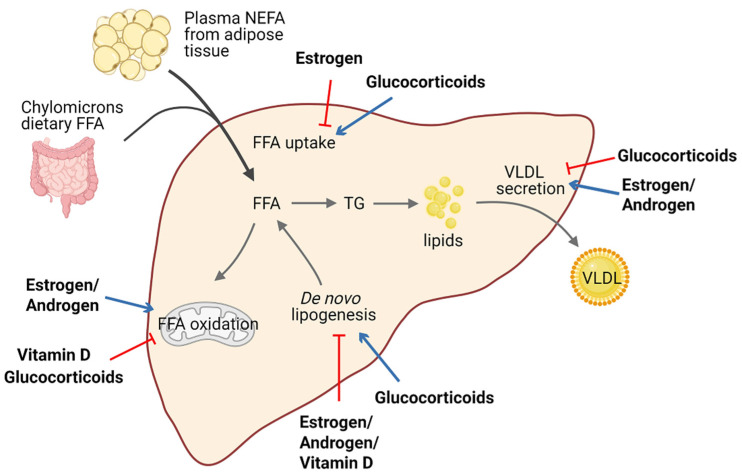
Schematic view of the roles of steroid hormones in hepatic lipid homeostasis. Steroid hormones have the ability to regulate lipid homeostasis via alteration of multiple major metabolic fates of hepatic FFAs. Blue arrows indicate stimulatory activity and red arrows indicate inhibitory activity. Abbreviations: free fatty acid (FFA); non-estesterified fatty acid (NEFA); triglyceride (TG); very-low-density lipoprotein (VLDL).

Lipid homeostasis is fine-tuned through four major pathways: uptake of circulating lipids, de novo lipogenesis, fatty-acid oxidation, and export of lipids in VLDL [69]. Hepatic steatosis occurs when there is an imbalance between lipid acquisition and lipid disposal. Increasing data have shown that steroid hormones are involved in hepatic steatosis by modulating these processes (Figure 2). We summarize steroid hormone-mediated effects of hepatic steatosis in animal models in Table 1.

It is well established that estrogen and ERs are involved in the pathogenesis of NAFLD. For example, ERα-deficient mice have disrupted lipid metabolism, such as decreased fatty-acid oxidation and increased de novo lipogenesis, leading to elevated lipid accumulation in the liver compared with normal mice [70,71,72]. Similarly, downstream blocking of ERα contributed to higher visceral fat accumulation and reduced energy expenditure in female mice [34,70,73]. Meanwhile, E2 treatment promoted fatty-acid oxidation in the liver by increasing the expression of the fatty-acid transport protein, carnitine palmitoyltransferase 1 (CPT-1) [34]. In ovariectomized (OVX) female mice, estrogen replacement improves insulin sensitivity and facilitates the VLDL-mediated export of lipids from the liver by increasing hepatic VLDL-TG production [74]. Interestingly, long-term therapy of tamoxifen, a selective estrogen receptor modulator, can increase the risk of NAFLD in breast cancer patients [75].

**Table 1:** Summary of steroid hormones effects on hepatic steatosis. ↑ increased; ↓ decreased.

Steroid Hormones	Model(s) Used	Major Phenotypes Examined
Estrogen	- Female ERα-deficient mice fed HFD for 10 weeks [71] - Male hepatic ERα-deficient mice fed HFD for 12 weeks [72] - OVX mice treated with E2 and fed HFD for 6 weeks [74]	- Liver weight, hepatic steatosis, and ALT level ↑ - Hepatic steatosis and insulin resistance ↑ - Hepatic steatosis and insulin- mediated suppression of VLDL secretion ↓
Androgen	- Male hepatic AR-deficient mice fed HFD for 8 weeks [76]	- Body weight, hepatic steatosis, and insulin resistance ↑
Glucocorticoid	- *db*/*db* mice treated with GC shRNA for 14 days [77] - SD rats treated with exogenous corticosterone and fed HFD for 16 days [78]	- Hepatic steatosis and genes critical for lipid storage and transport ↓ - Hepatic steatosis, uptake of FA into liver, and ALT level ↑
Mineralocorticoid	- Myeloid MR-deficient *ob*/*ob* mice [79] - Aldosterone synthase-deficient mice fed HFD for 12 weeks [80]	- Hepatic steatosis, lipogenesis, and insulin resistance ↓ - HFD-feeding-induced hepatic steatosis ↓
Vitamin D	- C57BL6 mice fed a high-fat/ high-sucrose diet followed by treatment with vitamin D for 15 weeks [81] - SD rats fed HFD followed by treatment with vitamin D for 16 weeks [82]	- Hepatic steatosis and hepatic de novo lipogenesis ↓ - Liver weight, hepatic steatosis, and ALT level ↓

A meta-analysis revealed that lower serum testosterone levels are associated with men with NAFLD [83,84]. Testosterone deficiency in men displayed an increased accumulation of visceral adipose tissue and insulin resistance, which are factors favoring the development of hepatic steatosis [85]. Similar results were confirmed in hepatic AR-deficient mice, indicating that AR might play a role in the suppression of NAFLD [76,86]. Indeed, androgen/AR signaling was revealed to suppress fatty-acid synthesis by decreasing the expression of sterol regulatory element-binding protein (SREBP) and to induce insulin sensitivity by modulating phosphoinositide-3 kinase activity [24].

Conversely, glucocorticoids drive the expression of lipogenic genes including fatty-acid synthase (*FASN)* and acetyl-coA carboxylase 1 (*Acaca*) [87], stimulate de novo lipogenesis, and block VLDL secretion, thus resulting in hepatic steatosis [88]. Corticosterone, administered to rodent models to increase basal glucocorticoids levels, also induces an increase in Cluster Determinant 36 (CD36) expression that facilitates fatty-acid uptake, favoring the progression of hepatic steatosis [78]. Of note, patients with glucocorticoid excess may develop hepatic steatosis, as well as obesity and insulin resistance, in a significant proportion of cases [89,90]. Moreover, fatty liver development also represents a typical side-effect of long-term systemic glucocorticoids treatment during anti-inflammatory therapy. Additionally, mice deficient in hepatic GR displayed lower hepatic TG content and elevated levels of ketone bodies in the serum, indicating that disruption of GR expression reduces steatosis in *db*/*db* animals partly by triggering hepatic fatty-acid oxidation and ketogenesis [77].

Macrophage-specific deficiency of MR protects mice from hepatic steatosis and insulin resistance through the ERα/HGF/Met pathway [79]. In addition, aldosterone deficiency attenuates high-fat feeding-induced hepatic steatosis, although the underlying mechanism remains elusive [80]. On the other hand, elevated aldosterone level seems to enhance hepatic FFAs via modulation of lipogenesis and lipolysis [91].

Vitamin D has been proven to protect against high-fat diet-induced fatty liver by acting on the expression of de novo lipogenesis-related genes, including *FASN* and *Acaca*, and fatty-acid oxidation-related genes such as the acetyl-coA oxidase (*Acox*) [81,82]. In humans, hepatic VDR expression is inversely correlated with steatosis severity [92]. Activation of VDR in hepatic macrophages via its cognate ligand has been uncovered to ameliorate steatosis and insulin resistance in experimental studies [93].

## 4. Role of Steroid Hormones in Hepatic Inflammation and Fibrosis

**Figure 3:**
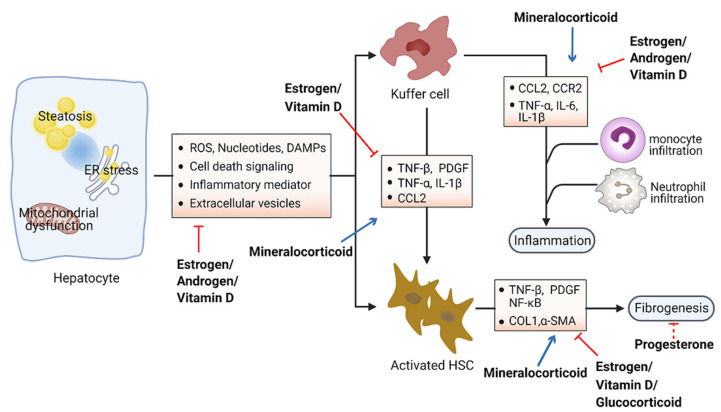
Schematic view of the roles of steroid hormones in NASH development. Steroid hormones are involved in guarding against hepatocellular damage, inflammation, and fibrosis in the pathological progression of NASH. Blue arrows indicate promoting activity and red arrows indicate inhibitory activity. Abbreviations: reactive oxygen species (ROS); damage-associated molecular patterns (DAMPs); tumor necrosis factor (TNF); platelet-derived growth factor (PDGF); interleukin (IL); C–C motif chemokine ligand 2 (CCL2); C–C motif chemokine receptor 2 (CCR2); nuclear factor kappa B (NK-kB); collagen I (COL1); α-smooth muscle actin (α-SMA).

Accumulation of toxic lipids leads to ROS, endoplasmic reticulum (ER) stress, cell death, release of damage-associated molecular patterns (DAMPs), and secretion of inflammatory mediators and extracellular vesicles, which stimulate an inflammatory response in Kupffer cells and a fibrotic response in hepatic stellate cells (HSC) [94,95]. The inflammatory process includes recruitment of macrophages and neutrophils to the site of injured tissue, followed by production of proinflammatory chemokines and cytokines such as interleukin 1 (IL-1) and tumor necrosis factor alpha (TNF-α) [96,97]. Furthermore, initiation of the inflammatory acute-phase response of the liver is induced by these cytokines together with IL-6 [98]. Liver inflammation can lead to fibrosis that may eventually induce cirrhosis [99]. Hepatic fibrosis is characterized by the accumulation of extracellular matrix. As a crucial driver of fibrosis, HSCs can be transdifferentiated into proliferative and fibrogenic myofibroblasts [100]. Profibrogenic cytokines such as vascular endothelial growth factor (VEGF), platelet-derived growth factor (PDGF), and transforming growth factor-β (TGF-β) are involved in the activation of HSCs and hepatic abnormal wound repair response [101,102,103]. Collectively, hepatocellular damage, inflammation, and fibrosis are the main characteristics of NASH [104,105]. Numerous studies have demonstrated the important effects of steroid hormones in the pathological progression of NASH (Figure 3). Here, we summarize steroid hormone-mediated effects on hepatic inflammation and fibrosis in animal models in Table 2.

Steroid hormones primarily display anti-inflammation and antifibrosis properties in the context of metabolic disorders. Estrogens have been found to directly suppress inflammatory processes in the liver. Women after menopause have decreased fatty-acid oxidation and increased lipogenesis in the liver, leading to the excessive accumulation of hepatic fat and culminating with inflammation [106,107]. Moreover, the generation of ROS and proinflammation cytokines was prevented by estrogen signaling in hepatocytes [108,109]. Estradiol treatment reduces hepatic inflammation in the animal model of NASH induced by methionine and a choline-deficient diet [110]. Furthermore, female mice with estrogen deficiency induced by OVX operation were observed with enhanced levels of macrophages infiltration and proinflammatory cytokines in the liver, including TNF-α, IL-6, C–C motif chemokine ligand 2 (CCL2), and C–C motif chemokine receptor 2 (CCR2) [111]. Hepatic fibrosis has also been shown to be associated with estrogen level. The risk of liver fibrosis increased significantly in a zebrafish experiment with ovarian senescence [112]. Similarly, a long duration of estrogen deficiency poses a high risk of hepatic fibrosis among postmenopausal women with NAFLD [113]. However, postmenopausal estrogen therapy may be considered for preventing the occurrence of liver fibrosis [114]. OVX female mice fed with a high-fat and high-cholesterol diet had obvious liver fibrosis with upregulated expression of collagen I α1, which could be improved by estrogen replacement therapy [109]. Additionally, β-LGND2, an ER-β-selective agonist, partially prevented inflammatory cell infiltration and liver fibrosis, providing a therapeutic benefit in NASH [115].

It has been shown that testosterone may increase the level of anti-inflammatory cytokines and reduce the level of proinflammatory cytokines, thereby exerting anti-inflammatory properties in patients with diabetes mellitus or coronary artery disease [116]. In experimental animal models of NAFLD, androgens were shown to be able to prevent NASH progression by acting on proinflammatory cytokines such as TNF-α and IL-6 [117].

Although increasing levels of progesterone have been associated with the development of systemic insulin resistance [118], little is known regarding the role of serum progesterone in NAFLD. Early studies have shown that treatment with progesterone in rabbits exerts a protective effect on hepatocytes against vacuolization and inflammation, accompanied by a low level of fibrosis [119].

Glucocorticoid hormones are considered the most widely used anti-inflammatory drugs. However, the effects of glucocorticoids on hepatic inflammation are still unclear, particularly in the progression of NAFLD. One study suggested that glucocorticoids may suppress the development of liver inflammation, considering that the perivascular infiltration of small mononuclear cells was diminished in the liver of mice following treatment with dexamethasone, a synthetic GR ligand [120]. Meanwhile, glucocorticoids suppress fibrotic gene expression including collagen I α1/2 by activating GR, which differentially regulates liver injury and hepatic fibrosis in HSCs or immune cells [120].

**Table 2:** Summary of the effects of steroid hormones on hepatic inflammation and fibrosis. ↑ increased; ↓ decreased.

Steroid Hormones	Model (s) Used	Major Phenotypes Examined
Estrogen	- OVX mice fed HFD and high-fructose water for 12 weeks [111] - OVX mice fed a high-fat and high-cholic-acid diet for 6 weeks [109] - Old female zebrafish fed a high-calorie diet for 24 weeks [112] - Orchidectomized C57/BL6 mice treated with estradiol benzoate-fed MCD for 4 weeks [110] - Male C57BL6 mice treated with β-LGND2 and fed HFD for 10 weeks [115]	- Hepatic inflammation and fibrosis, ALT level and ballooning degeneration ↑ - Liver fibrosis, inflammation, and hepatocyte ballooning degeneration ↑ - Liver fibrosis, IL-6, and TNF-β ↑ - Hepatic inflammation, MyD88, and IL-6 ↓ - Hepatic steatosis and insulin resistance ↓
Androgen	- Orchidectomized male SD rats treated with dihydrotestosterone and fed HFD for 75 days [117]	- Portal inflammation, TNF-α, and IL-6 ↓
Progesterone	- Hepatic fibrosis model of New Zealand male rabbits treated with progesterone for 180 days [119]	- Liver fibrosis, fat metamorphosis, and inflammatory infiltrate ↓
Glucocorticoid	- Immune cell-specific GR-knockout mice treated with CCl4 and dexamethasone for 6 weeks [120] - HSC-specific GR-knockout mice treated with CCl4 and dexamethasone for 6 weeks [120]	- Inflammation and monocyte recruitment ↓ - Hepatic fibrosis and fibrotic gene expression ↓
Mineralocorticoid	- C57BL6 mice fed HFFD mixed with eplerenone for 12 weeks [121] - Male C57BL6 mice fed a CDAA diet for 22 weeks with eplerenone [122] - Male SD rats treated with aldosterone for 4 weeks [123]	- Lipid accumulation, lobular inflammation, and collagen deposition ↓ - Hepatic fibrosis, steatosis, and inflammation ↓ - Hepatic fibrosis, oxidative stress, and DNA double-strand breaks ↑
Vitamin D	- Vitamin D-deficient SD rats fed WD for 10 weeks [124] - CDAA diet-induced rat NASH model with phototherapy for 6 or 12 weeks [125]	- Foci of lobular inflammation and ballooning degeneration ↑ - Collagen fibrosis, insulin and leptin resistance, inflammation, and HSC activation ↓

Aldosterone was observed to promote liver inflammation. ROS production can be stimulated by aldosterone in several tissues [126]. Blockade of aldosterone interaction with MR by eplerenone impeded macrophage infiltration and suppressed the expression of TNF-α and multiple copies in T-cell lymphoma-1 (MCT-1) in Kupffer cells, consequently ameliorating the development of NASH in mice [121]. Unlike the other steroids, mineralocorticoids foster the development of fibrosis by upregulating serum- and glucocorticoid-inducible kinase 1 (SGK1), which increases NF-κB, thus promoting fibrosis [127]. Additionally, aldosterone treatment could lead to liver fibrosis in male rats independent of blood pressure via promoting the expression of several fibrosis-associated genes (e.g., TGF-β, α-SMA) [123]. Furthermore, the expression of MR in hepatic stellate cells correlates with inflammation and fibrosis development in choline-deficient and amino-acid-defined diet-induced NASH [122]. Specific MR blockade with eplerenone effectively ameliorated histological steatosis and hepatic fibrosis in a mouse model of NASH. These data provide the basis for therapeutic exploitation of MR blockade for treatment of NASH [122].

Hepatic inflammation can also be affected by vitamin D through downregulation of the expression of Toll-like receptors on Kupffer cells [128]. Vitamin D deficiency causes upregulation of inflammation and oxidative stress genes [124]. Furthermore, restoration of vitamin D could effectively improve TNF-α-modulated immunological abnormalities in a diet-induced steatohepatitis rat model [125]. Moreover, serum vitamin D was significantly decreased in NAFLD patients with advanced liver fibrosis, suggesting that vitamin D might be associated with the progression of liver fibrosis [129]. Further study showed that vitamin D plays an antifibrotic role by suppressing the activation of the HSC-mediated TGF-β signaling pathway, inhibiting the accumulation of profibrotic extracellular matrix proteins [130] and the expression of profibrotic genes including collagen and α-smooth muscle actin (α-SMA) [131,132].

## 5. Conclusions and Perspective

Steroid hormones bound to cognate receptors perform diverse functions in lipid metabolism, modulating hepatic steatosis, as well as inflammation and fibrosis. In particular, the physiological changes can be made via the relevant gene expressions of lipid metabolism being affected by receptors directly or indirectly [63,117]. Specifically, estrogens, androgens, and vitamin D and their corresponding receptors play anti-steatosis, anti-inflammatory, and antifibrosis roles, while glucocorticoids play pro-steatosis, anti-inflammatory, and antifibrosis roles, and aldosterone plays pro-steatosis, proinflammation, and pro-fibrosis roles.

The complexity of NAFLD is presented not only in its association with other clinical conditions which include intricate organ crosstalk but also in its various and enigmatic biochemical mechanisms. Indeed, the increasing fatty acids from lipolysis of fat adipose tissue, the proceeding de novo lipogenesis, and the inhibition of β-oxidation of fatty acid in the mitochondria and inflammation from liver injury cause abnormal hepatic lipid accumulation and, eventually, NAFLD development [13]. Considering the existing differential biological functions of each steroid hormone under physiological conditions, especially related to these metabolism pathways, it is not surprising that steroid hormones have major impacts on the pathogenesis of NAFLD.

A number of studies on the general adult population have reported a higher prevalence of NAFLD in men than women [133,134,135]. Interestingly, the prevalence of NAFLD becomes more common in aged women [134,136,137,138]. Specifically, the prevalence of NAFLD was significantly higher in postmenopausal obese women (60.2%) than in premenopausal obese women (42.9%) [139]. The prevalence of NAFLD in men and postmenopausal women compared with premenopausal women highlights the nature of the sex disparity of NAFLD influenced by sex steroid hormones [140,141]. Indeed, recent studies have confirmed that estrogen or androgen (and their receptors) is able to decrease the incidence of NAFLD in women or men, respectively. Understanding the molecular mechanisms that promote the sex difference in NAFLD development might help pave ways to developing better therapeutic strategies.

Although steady progress has been disclosed in the pathogenesis of NAFLD, significant challenges still exist in the development of a therapeutic agent. This review summarized the action of several steroid hormones on NAFLD, not only providing the molecular mechanisms underlying this action, but also implicating the potential strategies for treating NAFLD in the future, for example, the development of novel steroid hormone analogues and cautious application of HRT based on the effects of sex steroid hormones in the sex disparity of NAFLD. Furthermore, several recent studies have provided evidence for the roles of steroid hormones in NAFLD. Specifically, it has been shown that total testosterone is inversely related to NAFLD in men, and the previously increased use of testosterone replacement may be encouraged if more exploration studies could be warranted [142,143]. Another recent study implied that vitamin D supplementation has no effect in patients with NAFLD since there are no changes in lipid profile or liver enzymes; however, when combined with vitamin E, calcium, or omega-3 fatty-acid supplementation, potential benefits emerged in those patients [144]. Nevertheless, new therapeutic strategies may be required to reduce the impact of NAFLD on modern society.
